# Family with sequence similarity 83, member A (FAM83A) inhibits ferroptosis via the Wnt/β-catenin pathway in lung squamous cell cancer

**DOI:** 10.1038/s41420-024-02101-4

**Published:** 2024-07-20

**Authors:** Cong Wang, Jing Zhang, Hongjiao Wang, Ruixue Chen, Ming Lu

**Affiliations:** 1 Department of Pharmaceutics, Key Laboratory of Chemical Biology (Ministry of Education), NMPA Key Laboratory for Technology Research and Evaluation of Drug Products, School of Pharmaceutical Sciences; Department of Radiation Oncology, Qilu Hospital of Shandong University, Cheeloo College of Medicine, Shandong University, Jinan, China; 2 Department of Drug Inspection, Tai’an Institute For Food And Drug Control (Tai’an Fiber Inspection Institute), Tai’an, China; 3 Department of Radiation Oncology, Qilu Hospital of Shandong University, Cheeloo College of Medicine, Shandong University, Jinan, China; 4 Department of Encephalopathy (II), Xintai Hospital of Traditional Chinese Medicine, Tai’an, China; 5 Department of Thoracic Surgery, Qilu Hospital, Cheeloo College of Medicine, Shandong University, Jinan, China

**Keywords:** Outcomes research, Prognostic markers, Translational research

## Abstract

The function of Family With Sequence Similarity 83, Member A (FAM83A) in lung squamous cell carcinoma (LUSC) is largely unknown. Here, we detected its prognostic and regulation roles in LUSC. Bioinformatics methods were applied initially to predict the expression level and prognostic value of FAM83A mRNA in LUSC. In vitro experiments, such as western blot, colony formation and cell viability assay, lipid Reactive oxygen species (ROS), malondialdehyde (MDA), reduced glutathione (GSH)/oxidized glutathione disulfide (GSSG), and 4-hydroxy-2-nonenal (4-HNE) assay, were used to investigate its mechanism. In vivo experiments were further conducted to validate the mechanism. Results from TCGA and Oncomine databases revealed significantly higher FAM83A mRNA expression levels in LUSC than in normal lung tissue. TCGA and GEO databases and our database revealed that FAM83A expression level was an independent prognostic factor for both overall survival and progression-free survival. Besides, FAM83A was significantly associated with a higher ability of growth and clonogenicity. Mechanistically, in vitro and in vivo experiments revealed that FAM83A could promote LUSC cell growth by inhibiting ferroptosis via activating the Wnt/β-catenin signaling pathway. The rescue experiment demonstrated that inhibition of the Wnt/β-catenin pathway counteracted the function of FAM83A. FAM83A is overexpressed in LUSC and could serve as a prognosis prediction biomarker for LUSC. FAM83A promotes LUSC cell growth by inhibiting ferroptosis via activating the Wnt/β-catenin signaling pathway, which provides a new potential therapeutic target for LUSC treatment.

## Background

Lung cancer is the leading cause of cancer-related mortality, with a higher global incidence than any other cancer type. It accounts for more than 10% of the total cancer incidence worldwide [1]. Lung squamous cell carcinoma (LUSC) is a distinct histologic subtype of non-small cell lung cancer (NSCLC), accounting for about 30% of cases [2]. LUSC is challenging to treat because of specific patient and disease characteristics, including older age and advanced disease at diagnosis and a higher incidence of comorbidities such as chronic obstructive pulmonary disease and heart disease. LUSC is usually centrally located, typically arising in the proximal bronchi, and as a consequence, it is more likely to invade larger blood vessels [3]. Recently, therapeutic options for the treatment of lung cancer have emerged through better understanding of the molecular mechanisms of tumor formation and progression. However, few inroads in targeted therapy and immune checkpoint inhibitor therapy have been made for LUSC [4], and chemotherapy remains the primary treatment for patients with advanced LUSC. Current 5-year survival estimates in LUSC range from 73% in stage IA disease to 13% in stage IV disease [5]. Future prognostication of outcomes in LUSC will likely be based on a combination of TNM stage and molecular tumor profiling to yield more precise, individualized survival estimates and treatment algorithms.

Family with sequence similarity 83, member A (FAM83A), also known as BJ-TSA-9 [6], is located on chromosome 8, locus q24.13, and spans 27 566 base pairs [7]. Recently, a number of studies have shown that dysregulated FAM83A is a potential biomarker in various cancers, including breast cancer, hepatocellular carcinoma, and lung adenocarcinoma [7–12]. It has been reported that FAM83A promotes the progression of lung adenocarcinoma by regulating the Wnt and Hippo [8] and ERK and PI3K/Akt/mTOR pathways [13], making it a promising therapeutic target. Besides, the circ-ZKSCAN1/miR-330-5p/FAM83A feedback loop plays an important role in promoting the progression of NSCLC [14]. However, the exact expression level of FAM83A in LUSC and its clinical prognostic value remains unknown. Therefore, in the present study, we used bioinformatics methods initially to predict the expression levels of FAM83A mRNA in LUSC compared with normal lung tissues. Then, immunohistochemical (IHC) staining was used to examine the FAM83A protein expression level and its prognostic value in LUSC. Interestingly, we found the typical feature of ferroptosis in FAM83A-knockdown cell lines. Ferroptosis, a unique modality of cell death driven by iron-dependent phospholipid peroxidation, is regulated by multiple cellular metabolic pathways, including redox homeostasis, iron handling, mitochondrial activity, and metabolism of amino acids, lipids, and sugars, in addition to various signaling pathways relevant to disease. However, the relationship between FAM83A and ferroptosis remains unclear. Here, we aimed to clarify the role and mechanism of FAM83A in the proliferation and ferroptosis regulation of LUSC cells, so as to provide evidence for new potential therapeutic targets for LUSC.

## Results

### FAM83A mRNA is upregulated in LUSC tissues

**Fig. 1:**
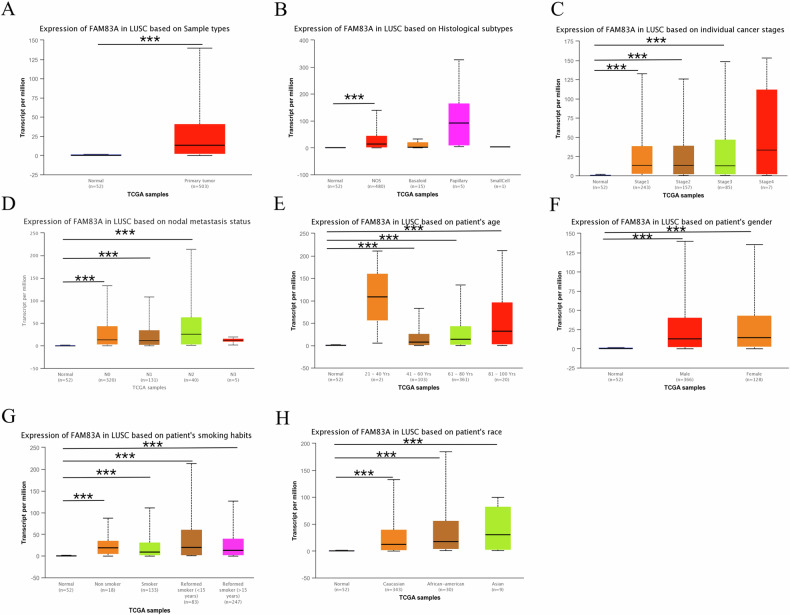
FAM83A mRNA expression level in LUSC in the TCGA database. **A** FAM83A mRNA levels in LUSC tissue vs. normal lung tissue. **B**–**H** FAM83A mRNA expression levels stratified by histologic subtype, individual cancer stage, nodal metastasis stage, patient’s age, gender, smoking habit, and race.

**Fig. 2:**
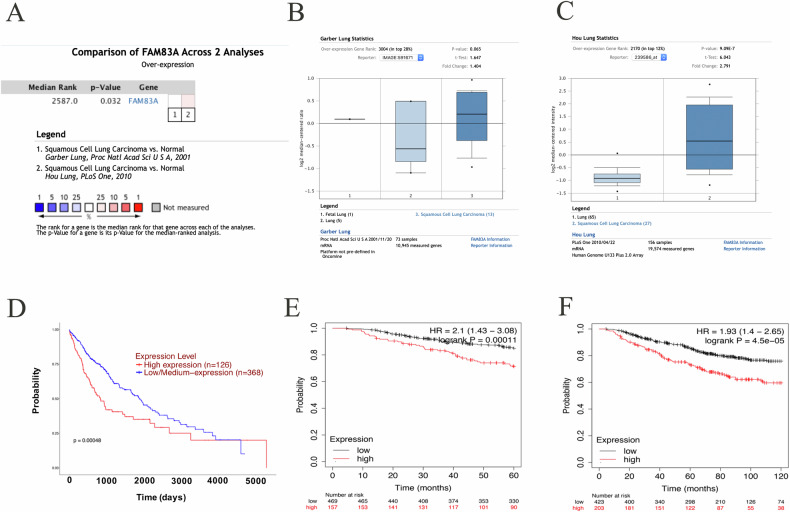
FAM83A mRNA level and prognostic prediction value in LUSC predicted by the online database. **A** Meta-analysis of the two datasets on FAM83A mRNA levels in LUSC tissue vs. normal lung tissue in the Oncomine database. **B**, **C** FAM83A mRNA expression level in the Garber Lung (GSE number: GSE3398) and Hou Lung (GSE number: GSE19188) datasets. **D** Overall survival (OS) curves of LUSC patients based on the differential expression levels of FAM83A mRNA (low vs. high) in the TCGA database. **E** 5-year and **F** 10-year OS curves of FAM83A mRNA prognostic curve based on the Kaplan–Meier database.

A total of 555 samples (503 cancerous tissues and 52 normal tissues) from TCGA database were included. We found that FAM83A mRNA was significantly upregulated in LUSC tissues compared with normal tissues (median value: 13.464 transcripts per million vs. 0.411 transcripts per million, *P* < 0.001; Fig. 1A). In subgroup analysis based on histologic subtype, FAM83A mRNA was upregulated in LUSC–not otherwise specified (NOS) type but not in basaloid, papillary, and small cell types. Besides, FAM83A was upregulated in LUSC tissue of stages 1, 2, and 3 compared with normal tissues, as well as in LUSC tissues of N0, N1, and N2. Compared with normal controls, FAM83A mRNA was overexpressed in all patient subgroups based on age, race, gender, and smoking habit (all *P* < 0.001, Fig. 1E–H), except for the subgroup of patients aged 21–40 years. By searching the Oncomine database, a total of two GEO-sourced datasets were found. Meta-analysis revealed that FAM83A mRNA levels were significantly higher in LUSC tissues than in normal lung tissues (*P* = 0.032, Fig. 2A). Moreover, as shown in Fig. 2B, C, the results from the datasets were consistent with the findings of the abovementioned meta-analysis. To verify the expression level of FAM83A protein in LUSC tissues, we performed IHC in 132 pairs of LUSC and paracancerous tissues. No significant relationship was found between FAM83A expression and clinicopathological features such as age, gender, smoking, drinking, T stage, N stage, and differentiation (Table 1). FAM83A was overexpressed in 78 (59.1%) cancerous tissues and 19 (14.4%) paracancerous tissues (*P* < 0.001).

**Table 1:** Correlation of clinicopathological variables with FAM83A expression in LUSC samples.

Clinicopathological features	FAM83A overexpression		*P*^a^ value
	No (*n* = 54)	Yes (*n* = 78)	
Age			0.369
<65	25	30	
≥65	29	48	
Gender			0.279
Female	23	26	
Male	31	52	
Smoking			0.734
Never or light	38	57	
Heavy	16	21	
Drinking			0.135
Never or light	46	58	
Heavy	8	20	
Differentiation			0.497
Well	19	22	
Moderate	15	29	
Poor	20	27	
T stage			0.404
T1	20	22	
T2	23	33	
T3	11	23	
N stage			0.441
N0	18	19	
N1	13	23	
N2	18	23	
N3	5	13	

*P*^a^ Chi-square test. *LUSC* lung squamous cell cancer.

### The prognostic value of FAM83A in LUSC

**Fig. 3:**
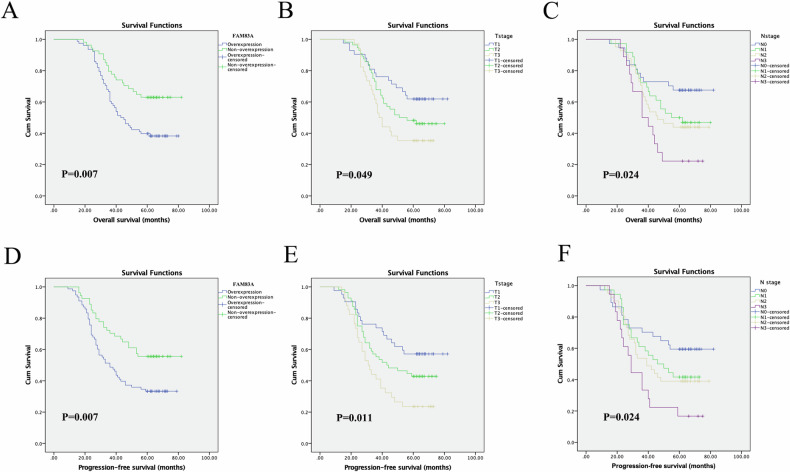
Kaplan–Meier analysis and log-rank test for 5-year OS and PFS of LUSC patients. **A**, **D** High FAM83A protein expression significantly predicted decreased OS and PFS. **B**, **C**, **E**, **F** T stage, and N stage were also associated with OS and PFS of LUSC patients.

Based on the TCGA database, high level of FAM83A mRNA was significantly associated with poorer OS of LUSC patients (*P* = 0.00048, Fig. 2D). We further browsed the Kaplan–Meier database, showing that higher FAM83A mRNA expression level also predicted poorer 5- and 10-year OS (both *P* < 0.001) (Fig. 2E, F). Next, we analyzed the prognostic value of FAM83A protein level using our own data. Of the 132 patients who provided FFPE cancer tissues, 64 (48.5%) survived more than 5 years after pneumonectomy, and 68 (51.5%) died during the follow-up period. The mean OS time for all of the patients was 50.5 ± 17.7 months (range, 15–82 months), and the mean PFS time was 45.5 ± 20.6 months (range, 9–82 months). Kaplan–Meier analyses followed by the log-rank test were performed to calculate the effect of the clinicopathological factors on the OS and PFS rates. High FAM83A protein expression was significantly associated with decreased 5-year OS (*P* = 0.007) and PFS (*P* = 0.007) (Fig. 3, Table 2). Furthermore, via multivariate analysis, FAM83A expression level was shown to be an independent prognostic factor for OS (hazard ratio [HR], 2.160; 95% confidence interval [CI], 1.374–3.396, *P* = 0.006), as well as PFS (HR, 2.266; 95% CI, 1.367–3.756, *P* = 0.002; Table 2). Besides, T and N stages were significant prognostic indicators for PFS (*P* = 0.008 and *P* = 0.033, respectively).

**Table 2:** Univariate and multivariate analyses of prognostic variables for LUSC patients.

	OS Univariate analysis		OS Multivariate analysis		PFS Univariate analysis		PFS Multivariate analysis	
Variables	HR (95% CI)	*P* value	HR (95% CI)	*P* value	HR (95% CI)	*P* value	HR(95% CI)	*P* value
Gender								
Female	Ref.	–			Ref.	–		
Male	0.935 (0.569–1.539)	0.793			0.958 (0.599–1.533)	0.858		
Age								
<65 years old	Ref.	–			Ref.	–		
≥65 years old	1.024 (0.632–1.660)	0.923			1.027 (0.650–1.624)	0.908		
Smoking								
Never or light	Ref.	–			Ref.	–		
Heavy	1.589 (0.961–2.627)	0.071			0.648 (0.401–1.047)	0.076		
Drinking						0.437		
Never or light	Ref.	–			Ref.	–		
Heavy	1.319 (0.753–2.311)	0.333			0.809 (0.472–1.388)	0.442		
Differentiation		0.240				0.490		
Poor	Ref.	–			Ref.	–		
Median	1.606 (0.891–2.894)	0.115			1.319 (.766–2.274)	0.318		
Well	1.143 (0.609–2.146)	0.677			0.993 (0.557–1.772)	0.982		
T stage		0.049*		0.080		0.011*		0.008*
T1	Ref.	–	Ref.	–	Ref.	–	Ref.	–
T2	1.557 (0.848–2.858)	0.153	1.277 (0.688–2.373)	0.438	1.522 (0.853–2.713)	0.155	1.243 (0.689–2.245)	0.470
T3	2.202 (1.154–4.204)	0.017*	2.057 (1.065–3.971)	0.032*	2.494 (1.363–4.565)	0.003*	2.446 (1.323–4.523)	0.004*
N stage		0.024*		0.053		0.024*		0.033*
N0	Ref.	–	Ref.	–	Ref.	–	Ref.	–
N1	1.723 (0.836–3.551)	0.140	1.741 (0.832–3.644)	0.141	1.553 (0.800–3.015)	0.193	1.605 (0.817–3.154)	0.170
N2	1.981 (0.985–3.986)	0.055	1.903 (0.936–3.869)	0.076	1.762 (0.928–3.347)	0.083	1.717 (0.895–3.294)	0.104
N3	3.334 (1.535–7.242)	0.002*	3.076 (1.380–6.856)	0.006*	3.058(1.487–6.287)	0.002*	3.079 (1.452–6.532)	0.003*
FAM83A								
Low	Ref.	–	Ref.	–	Ref.	–	Ref.	–
High	2.058 (1.220–3.471)	0.007*	2.160 (1.374–3.396)	0.006*	1.940 (1.194–3.151)	0.007*	2.266 (1.367–3.756)	0.002*

^*^P < 0.05; *OS* overall survival, *PFS* progression-free survival

### FAM83A promotes LUSC proliferation

**Fig. 4:**
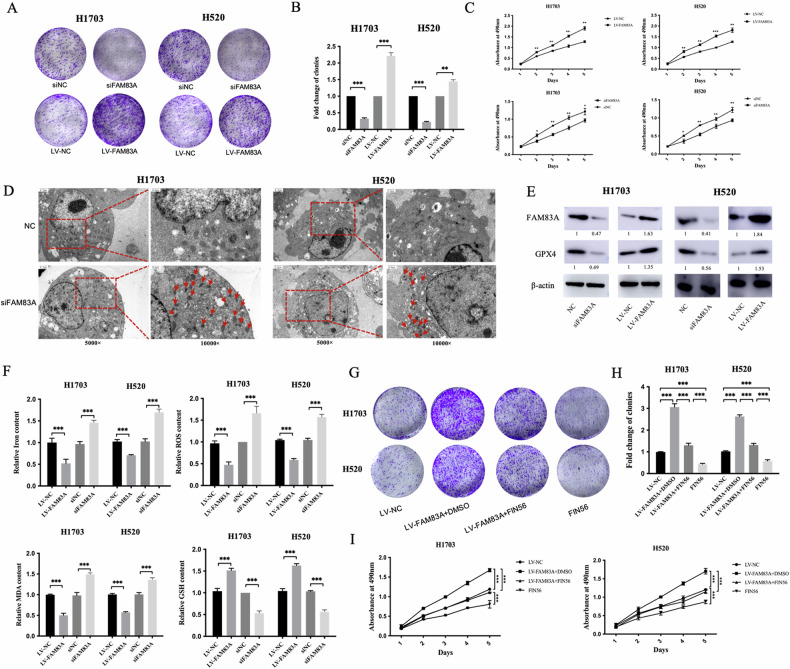
FAM83A promoted LUSC cell proliferation via inhibiting its ferroptosis level. **A**–**C** Colony formation and CCK-8 experiments were conducted in FAM83A-overexpression and -knockout groups. FAM83A overexpression increased the colony formation and cell proliferation capacity, while FAM83A knockdown inhibited its colony formation and cell proliferation capacity. **D** The ferroptosis features shown by the electron microscopy after FAM83A knockdown. The mitochondrial volume decreased, the double-membrane density increased, and the mitochondrial ridge decreased or disappeared in the siFAM83A group. **E** Western blot revealed that FAM83A downregulation inhibited glutathione peroxidase 4 (GPX4) expression, while GPX4 upregulated in FAM83A overexpression group. **F** FAM83A overexpression inhibited ferroptosis, including decreasing Fe^2+^, ROS, and MDA content and increasing GSH level, whereas the siFAM83A group induced the opposite results. **G**, **H** Colony formation and **I** CCK8 experiments revealed that FAM83A overexpression increased LUSC proliferation, while FIN56 counteracted its function.

We further studied the effect of FAM83A on the proliferation of LUSC cells. Lentivirus transfection method was used for FAM83A upregulation, and plasmid was used for FAM83A downregulation. CCK8 test showed that after upregulation of FAM83A, the cloning formation capacity of H1703 and H520 cell lines significantly increased, whereas it was significantly decreased in the siFAM83A group (Fig. 4A, B, both *P* < 0.001). The same trend was observed in CCK-8 proliferation experiments (Fig. 4C).

We further explored the regulatory mechanism. Interestingly, electron microscopy revealed that the mitochondrial volume decreased, the double-membrane density increased, and the mitochondrial ridge decreased or disappeared in the siFAM83A group (Fig. 4D), which are typical features of ferroptosis [15]. To further determine the presence of ferroptosis in LUSC cells, we examined the expression of glutathione peroxidase 4 (GPX4), which is the essential protein for ferroptosis. We found that FAM83A downregulation inhibited GPX4 expression (Fig. 4E). Moreover, we analyzed the intracellular Fe^2+^ content, ROS, malondialdehyde (MDA), a lipid peroxide product, and glutathione (GSH), an important substrate of GPX4. We found that FAM83A overexpression could inhibit ferroptosis, including decreasing Fe^2+^, ROS, and MDA content and increasing GSH level, whereas the siFAM83A group showed the opposite results (Fig. 4F). FIN56, a ferroptosis inducer, was used to validate the role of FAM83A in ferroptosis. Colony formation and CCK8 experiments revealed that FAM83A overexpression increased the proliferation ability of LUSC, whereas FIN56 counteracted the function of FAM83A (Fig. 4G–I).

### FAM83A inhibits ferroptosis via the Wnt/β-catenin pathway

**Fig. 5:**
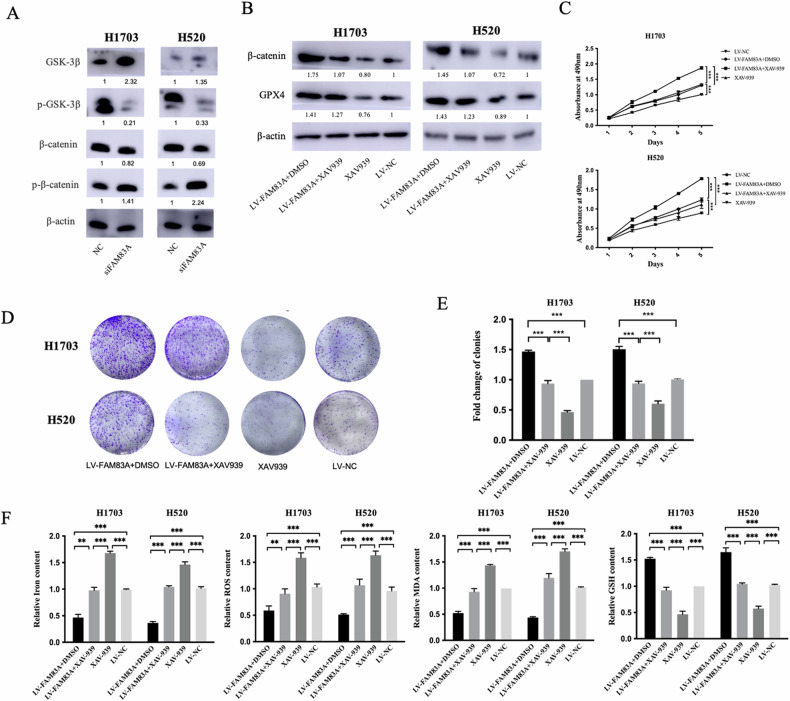
FAM83A inhibits ferroptosis via the Wnt/β-catenin pathway. **A** FAM83A knockdown increased the expression levels of GSK-3β and p-β-catenin, and decreased the expression levels of p-GSK-3β and β-catenin. **B** FAM83A overexpression increased the protein expression levels of β-catenin and GPX4, as well as the ability of cell growth (**C**) and clonogenicity (**D**, **E**), whereas inhibition of the Wnt/β-catenin (XAV-939) counteracted its function. **F** FAM83A inhibited ferroptosis (the decreased level of iron content, MDA, ROS, and increased GSH), whereas XAV-939 counteracted its effect.

It has been reported that the Wnt/β-catenin signaling pathway plays an important role in ferroptosis. The β-catenin/TCF4 transcription complex directly binds to the promoter region of GPX4 and induces its expression, resulting in the suppression of ferroptosis [16]. After regulation of FAM83A, we detected the protein levels of the Wnt/β-catenin pathway. We found that FAM83A knockdown could increase the expression level of GSK-3β, and decreased the expression level of p-GSK-3β (Fig. 5A), indicating that FAM83A could induce the activation of the Wnt/β-catenin pathway via increase the phosphorylation of GSK-3β. GSK3β phosphorylates β-catenin and results in the degradation of β-catenin, which inhibits the activity of the Wnt/β-catenin signaling pathway [17]. We arose the hypothesis that FAM83A could inhibit GSK3β function, which decreases the expression level of p-β-catenin and increased β-catenin expression. As a result, upregulated β-catenin could transport into the nucleus and promote the transcription of GPX4. To further verify the role of the Wnt/β-catenin pathway in FAM83A-mediated ferroptosis in LUSC, we used XAV939 to inhibit the transcription function of Wnt/β-catenin pathway. FAM83A overexpression increased the protein expression levels of β-catenin and GPX4, as well as the ability of cell growth and clonogenicity, whereas the rescue experiment demonstrated that inhibition of the Wnt/β-catenin pathway counteracted the function of FAM83A (Fig. 5B–E). As for ferroptosis-associated GSH, MDA, ROS, and iron content, we also found the same tendency (Fig. 5F).

**Fig. 6:**
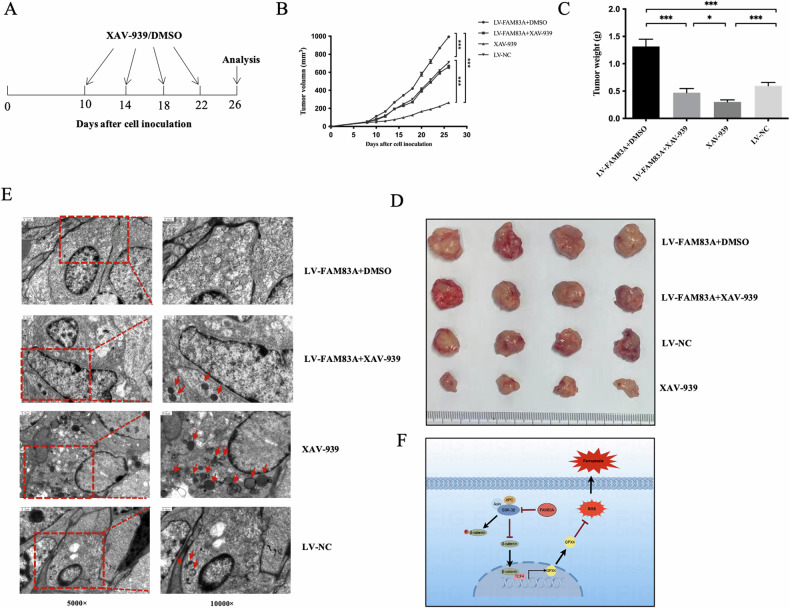
The inhibition of ferroptosis by FAM83A overexpression could be rescued via inhibiting wnt/β-catenin pathway in vivo. **A** Schedule of in vivo administration approach. **B** Tumor growth curves of tumor-bearing mice following different treatments. **C** Tumor weight in different groups at the study endpoint. **D** Tumor photographs of the sacrificed mice at the study endpoint. **E** The variation trend of ferroptosis observed via electron microscope analysis in four groups. **F** Schematic diagram of the biological role of FAM83A in ferroptosis regulation via the wnt/β-catenin pathway in lung squamous cell cancer.

Besides, in vivo experiments validated that FAM83A overexpression could inhibit ferroptosis and promote the tumor growth. At the same time, XAV-939 increased ferroptosis and inhibited tumor growth. In addition, the inhibition of ferroptosis by FAM83A overexpression could be rescued by wnt/β-catenin pathway inhibitor, XAV-939 (Fig. 6A–E). Taken together, FAM83A could promote LUSC cell growth by inhibiting ferroptosis via activating the Wnt/β-catenin signaling pathway.

## Discussion

LUSC remains largely incurable because of high flexibility of cancer cells and multiple regulatory feedback loops resulting in therapy resistance. Consequently, promising molecular targets are needed. It has recently been reported that FAM83A is highly upregulated in several cancer types. Its function in cancer cells is largely unknown; specifically, its role in LUSC remains unclear. To acquire a better understanding of FAM83A, we detected its expression and prognostic role using online databases and 132 pairs of FFPE samples in this study. Using the TCGA and GEO databases, we found that FAM83A mRNA was significantly upregulated in LUSC tissues compared with normal tissues. These findings correspond to those of previous studies that identified elevated amounts of FAM83A in various tumor tissues [11, 12, 18, 19]. Moreover, elevated expression of FAM83A mRNA negatively affected survival based on the TCGA and Kaplan–Meier databases. Next, to confirm the predictive results, IHC was used to investigate the expression levels of FAM83A protein. Consistent with the previous predictive findings [7, 9, 20], our multivariate analysis revealed FAM83A protein level as an independent prognostic factor for 5-year OS as well as PFS in LUSC patients. In summary, these findings suggest that FAM83A protein expression could serve as a prognosis biomarker of LUSC patients, and FAM83A might be an important target gene involved in the growth and metastasis of LUSC.

Recent studies revealed the mechanisms of FAM83A in various cancers. Zheng et al. [8] found that FAM83A promoted lung cancer progression by regulating the Wnt and Hippo signaling pathways. Hu et al. [13] found that FAM83A promoted tumorigenesis of NSCLC via the ERK and PI3K/Akt/mTOR pathways, making it a promising therapeutic target. Zhou et al. [21] found that co-expression of FAM83A and PD-L1 in tumor cells was a credible biomarker predictor for poor survival in resected lung adenocarcinoma patients. FAM83A may promote the expression of PD-L1 through the ERK signaling pathway, thereby causing immune escape of the tumor. In contrast to the overexpression level and oncogene roles of FAM83A in various cancers, Xu et al. [18] reported that FAM83A exerted a tumor‑suppressive role in cervical cancer by suppressing the expression of integrins, which may offer new insight into FAM83A’s regulatory mechanisms in cancer. In this study, we found that FAM83A inhibited ferroptosis via the Wnt/β-catenin pathway. It has been reported that the Wnt/β-catenin pathway induces GPX4 expression via the TCF4 transcription complex directly binding to the promoter region of GPX4, resulting in the suppression of ferroptotic cell death [16]. In the present study, overexpression of FAM83A in LUSC cell lines was significantly associated with higher ability of growth and clonogenicity. Mechanistically, FAM83A could promote LUSC cell growth by inhibiting ferroptosis via activating the Wnt/β-catenin signaling pathway. GSK3β phosphorylates β-catenin and results in the degradation of β-catenin, which inhibits the activity of the Wnt/β-catenin signaling pathway [17]. We found that FAM83A could inhibit GSK3β function, which decreases the expression level of p-β-catenin. As a result, upregulated β-catenin could transport into the nucleus and promote the transcription of GPX4. The rescue experiment demonstrated that inhibition of the Wnt/β-catenin pathway counteracted the function of FAM83A.

There are some limitations to this study. The number of histologic samples was small, which may have influenced the statistical analysis to some extent. More LUSC cell lines need to be used for the regulation mechanism validation.

## Conclusion

In conclusion, FAM83A is overexpressed in LUSC and could serve as a prognosis prediction biomarker for LUSC. FAM83A promotes LUSC cell growth by inhibiting ferroptosis via activating the Wnt/β-catenin signaling pathway. Suppression of FAM83A might be a potential strategy to inhibit LUSC proliferation via promoting their ferroptosis susceptibility.

## Methods and materials

### Cell lines

Lung squamous cell lines H1703 and H520 were purchased from Meisen Cell Technology Company (Zhejiang, PR China). RPMI-1640 (Gibco) with 10% fetal bovine serum (FBS) was used for cell culture in an incubator containing 5% CO_2_ at 37 °C.

### Reagents and antibodies

A ferroptosis inducer FIN56 (HY-103087) and a Wnt/β-catenin inhibitor XAV939 (HY-15147) were purchased from MedChemExpress company (USA). Anti–β-catenin (ET1601-5), anti–p-β-catenin (HA722316), anti–GSK3β (ET1607-71), anti–p-GSK3β (SY02-71), anti-GPX4 (JU11-31) antibodies, and anti–β-actin (EM21002) were purchased from Huabio company (China). Anti-FAM83A (20618-1-AP) polyclonal antibody was purchased from Proteintech (USA).

### Bioinformatics analysis

The TCGA (the comprehensive catalog of genomic abnormalities) data were analyzed and downloaded via UALCAN, a comprehensive and interactive web resource for analyzing cancer OMICS data (http://ualcan.path.uab.edu). The Oncomine database (https://www.oncomine.org) [22] was also screened to explore the differential expression levels of FAM83A between LUSC and normal groups. Additionally, Kaplan–Meier Plotter (http://kmplot.com) was used to draw the OS and PFS curves based on the GEO data.

### IHC staining

We collected 132 pairs of formalin-fixed paraffin-embedded (FFPE) cancerous and matched paracancerous LUSC tissues in 2012 from the Department of Pathology of Qilu Hospital. After deparaffinization and endogenous-peroxidase activity blocking, the slides were incubated with primary rabbit anti-FAM83A polyclonal antibody. Afterward, the slides were incubated with biotinylated secondary antibodies and streptavidin–peroxidase complex. The sections were incubated with PBS instead of the primary antibodies for negative controls. Finally, a 3,3′-diaminobenzidine solution was added, and the slides were counterstained with hematoxylin and mounted with neutral balsam. The sections were observed under a light microscope and independently scored by three investigators. The final score was calculated by multiplying the staining intensity (scored as follows: 0, no staining; 1, weak staining; 2, moderate staining; and 3, strong staining) by the percentage of positive cells (scored as follows: 0, 0–10% positive cells; 1, 10–25% positive cells; 2, 26–50% positive cells; 3, 51–75% positive cells; and 4, 76–100% positive cells). The final staining score was the sum of the staining intensity and percentage of positive cells. It was further graded as follows: 0–1, (−); 2–3, (+); 4–5, (++); and 6–7, (+++). The expression of FAM83A was divided into a non-overexpressed group (− or +) and an overexpressed group (++ or +++).

### Colony formation and cell viability assay

To perform colony formation experiments, cells (500 cells/well) were seeded in six-well plates and incubated for 10–14 days until cell colonies appeared, followed by fixing with methanol and staining with Giemsa.

Cell viability was measured using a CCK-8 reagent (MedChemExpress, USA). In short, cells were inoculated in 96-well plates and incubated using the specified treatment method. Subsequently, 100 μL of fresh medium containing 10 μL of CCK-8 solution was added to the cells and incubated at 37 °C and 5% CO_2_ for 2 h. Then, the absorbance was measured at 450 nm by spectrophotometry. The obtained values were normalized to blank holes, and the relative cell viability was normalized to the respective DMSO-treated holes.

### Lipid reactive oxygen species (ROS) assay

ROS kit (Beyotime Technology) was used for ROS level detection. The cells were plated on a 12-well plate (1 × 10^5^ cells/well). The medium was supplemented with 10 μM DCFH-DA and incubated at 37 °C and 5% CO_2_ for 30 min. The cells were collected by trypsin without EDTA, washed twice with PBS, and then suspended with 500 μL PBS. CytoFLEX Cytograph (Beyotime Technology) and CytExpert software (Beckman Coulter) were used for ROS analysis.

### MDA, reduced GSH/oxidized glutathione disulfide (GSSG) assay

The lipid peroxidation (MDA) assay kit (Beyotime Technology, China) was used to detect the relative concentration of MDA in accordance with the manufacturer’s instructions. TBA was added into the lysate to produce MDA–TBA adducts and quantified by colorimetry (OD = 532 nm). GSH level was measured using the GSH assay kit (Abcam, USA). The kit is based on an enzymatic cycling method in the presence of GSH and a chromophore. The reduction of the chromophore produces a stable product, which can be followed kinetically at 450 nm. Therefore, its absorbance is directly proportional to the amount of GSH in the sample.

### 4-hydroxy-2-nonenal (4-HNE) assay

The concentration of 4-HNE was evaluated by using a 4-HNE ELISA kit (Abcam, USA) in accordance with the manufacturer’s protocol.

### Cell transfection

We used the human FAM83A lentivirus targeting to upregulate FAM83A (LV-FAM83A) and the shRNA lentivirus targeting to knock down FAM83A (shFAM83A: 5′-GGA GUG UGG AAG GAG AGA UTT-3′) in accordance with a previous article [23]. The shRNA negative control lentivirus (shNC) was purchased from GeneChem Co., Ltd. (Shanghai, China).

### Western blot

RIPA buffer with phenylmethylsulfonyl fluoride was used for cell lysis. Total protein samples were mixed with SDS-PAGE loading buffer (Beyotime) and denatured at 95 °C for 10 min. The protein samples were then separated using SDS-PAGE (Beyotime) and transferred onto polyvinylidene difluoride membranes (Merck, Germany). The membranes were then blocked in milk at 5% w/v for 2 h and incubated with primary antibody at 4 °C overnight. After three washes with TBST, the membranes were treated with enzyme-labeled secondary antibody (CST) at room temperature for 2 h and detected using ECL Western Blot Substrate (Beyotime, China). Images were captured and analyzed using the ChemiDoc XRS+ System (Bio-Rad, Hercules, CA, USA).

### Animal experiments

All xenograft experiments were performed in accordance with the approved protocols of the Animal Experimentation Rules issued by the Chinese Government (Beijing, China). Five-week-old BALB/c-nude male mice were purchased from Beijing Sibeifu Biotechnology (Beijing, China). Mice were randomly assigned to four groups: (1) LV-FAM83A + DMSO, (2) LV-FAM83A + XAV-939, (3) LV-NC and (4) XAV-939. H1703 cells (1 × 10^7^) carrying the corresponding vector were injected subcutaneously into the axilla of the mice. When tumors reached a volume of ~150 mm^3^, XAV-939 was injected every 4 days. Tumor volume was measured every 2 days until the endpoint and calculated according to the formula volume = length × width^2^ × 1/2. Animal body weights were monitored every 2 days during the whole experiment. Then, the mice were sacrificed. Tumors were removed and collected via electron microscope analyses.

### Statistical analysis

The putative associations between clinical parameters and FAM83A were assessed by the Chi-square tests. Kaplan–Meier analysis was performed to estimate the survival curves in different subgroups, and the log-rank test (Mantel–Cox) was used to compare the curves. We then used Cox proportional-hazards model to identify the association of FAM83A expression level with survival and to estimate mortality HRs. The one-way ANOVA analysis of variance with Tukey’s post hoc test among multiple groups and Student’s t-test was used for statistical significance calculation. Statistical analyses were performed using SPSS statistical software (version 22.0) and GraphPad Prism 8.0.1. All P values were two-sided. *P* values < 0.05 were considered statistically significant.

### Supplementary information

SUPPLEMENTAL MATERIAL

## Data Availability

All online data are available from UALCAN (http://ualcan.path.uab.edu), Oncomine database (https://www.oncomine.org), and Kaplan–Meier Plotter (http://kmplot.com). Other data are available from the corresponding author.
