# Supplementary figures and images for: Family with sequence similarity 83, member A (FAM83A) inhibits ferroptosis via the Wnt/β-catenin pathway in lung squamous cell cancer
Source: Cell Death Discov. 2024 Jul 20;10:332. doi: 10.1038/s41420-024-02101-4 (PMC11271298; doi:10.1038/s41420-024-02101-4)

**Fig 4E**

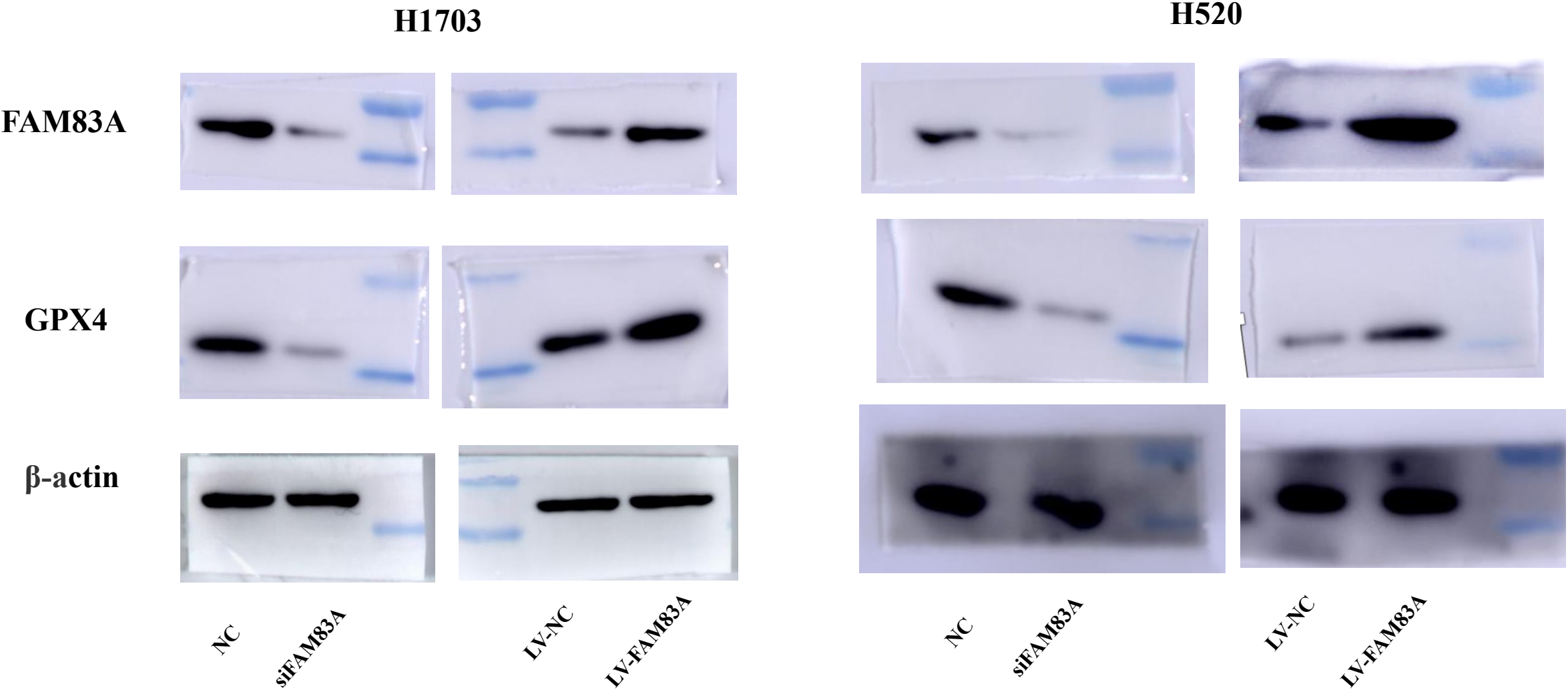

Fig 5A

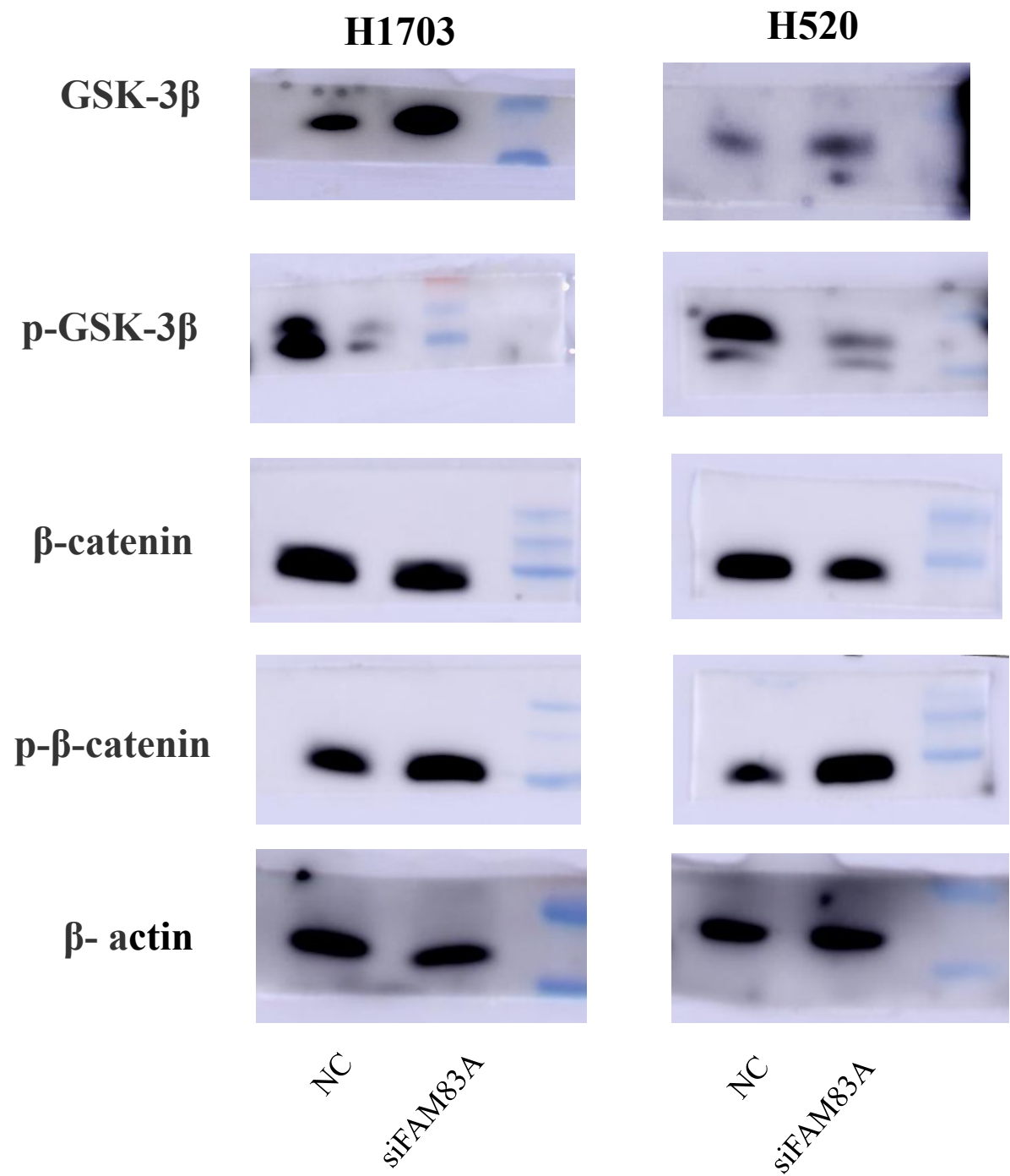

**Fig 5B**

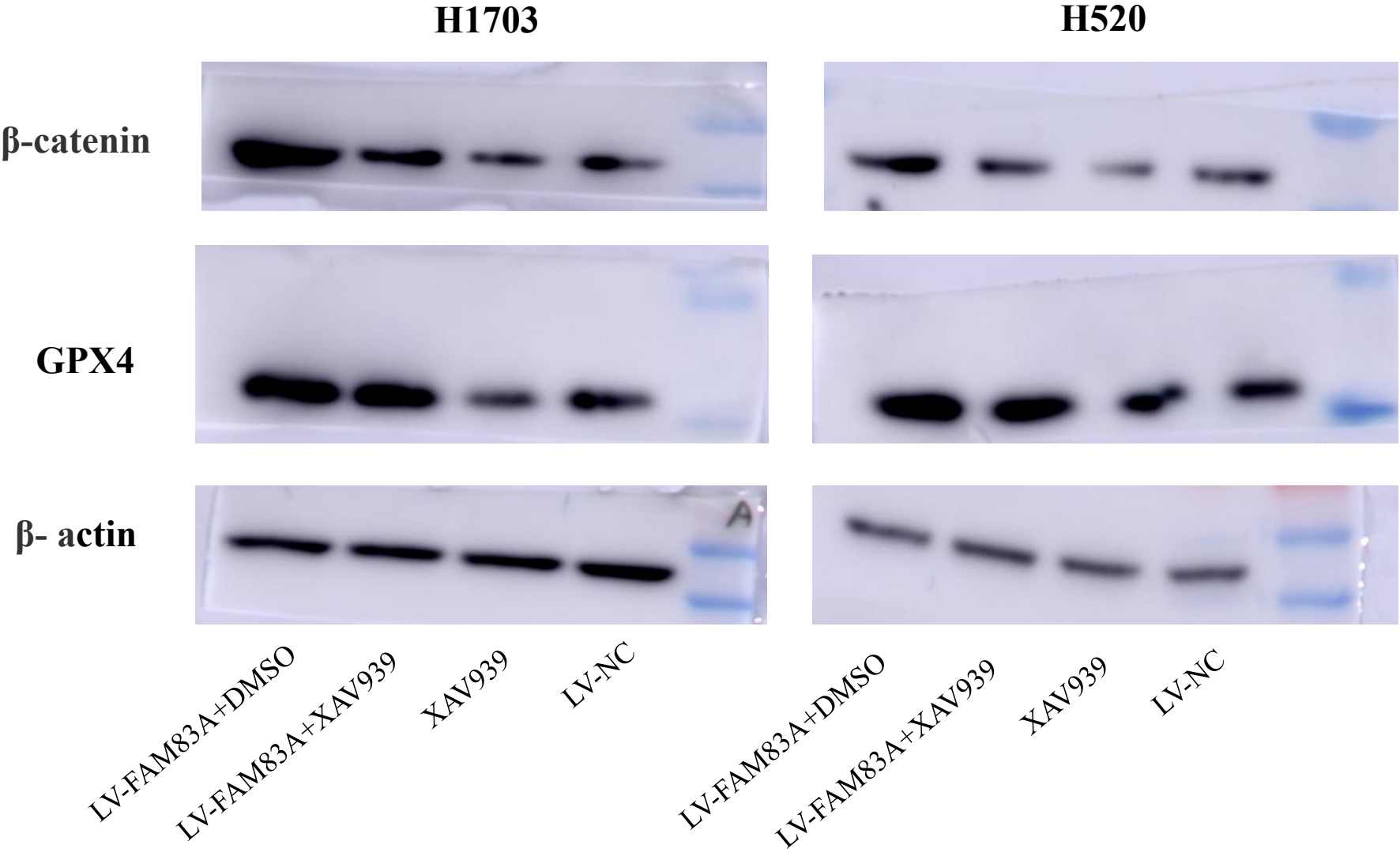

Supplement: Supplementary file 1 — SUPPLEMENTAL MATERIAL [file 41420_2024_2101_MOESM1_ESM.pdf]
